# Supplementary material for: Free amino acids accelerate the time-dependent inactivation of rat liver nucleotide pyrophosphatase/phosphodiesterase Enpp3 elicited by EDTA
Source: Amino Acids. 2024 Dec 6;57(1):1. doi: 10.1007/s00726-024-03431-4 (PMC11624235; doi:10.1007/s00726-024-03431-4)
Supplement: Supplementary file 2 — Supplementary file2 (PDF 130 KB) [file 726_2024_3431_MOESM2_ESM.pdf]

Supplementary Table S2 to

Free Amino Acids Accelerate the Time-Dependent Inactivation of Rat Liver Nucleotide Pyrophosphatase / Phosphodiesterase Enpp3 elicited by EDTA

By

Ana Romero, Guadalupe Cumplido-Laso, Ascensión Fernández, Javier Moreno, José Canales, Rui Ferreira, Juan López-Gómez, João Meireles Ribeiro, María Jesús Costas and José Carlos Cameselle

Table S2. Data used for the correlation analyses shown in main manuscript Fig. 9 and Fig. 10.

Amino acid	$k_{i(ap)}$ increase (%)	Molecular weight	Partial molar volume of whole amino acid (cm^3/mol)	Hydrophobicity average values of 12 different scales	α-Amino group pK_a	Amino acid-Zn^{2+} stability constant (25°C, μ 0.1) (L/mol)	Amino acid-Zn^{2+} stability constant (37°C, μ 0.15) (L/mol)
Alanine	270	89.09	60.54	-0.50	9.71	38018.94	34673.69
Arginine	100	174.20	127.42	1.59	9.00	12589.25	11220.18
Asparagine	270	132.12	78.00	0.72	8.73	33113.11	28183.83
Aspartic acid	430	133.10	74.80	1.37	9.66	741310.24	660693.45
Glutamine	170	146.14	–	0.72	9.00	–	16595.87
Glutamic acid	240	147.13	89.85	1.13	9.58	–	52480.75
Glycine	560	75.07	43.26	-0.09	9.58	91201.08	74131.02
Isoleucine	80	131.17	105.80	-1.20	9.60	–	25118.86
Leucine	110	131.17	107.77	-1.14	9.58	36307.81	32359.37
Lysine	70	146.19	108.50	1.57	9.16	12882.50	11481.54
Methionine	170	149.21	105.57	-0.87	9.08	23988.33	19952.62
Phenylalanine	210	165.19	121.50	-1.30	9.09	20417.38	16595.87
Proline	60	115.13	82.76	0.24	10.47	186208.71	134896.29
Hydroxyproline	170	131.13	84.49	–	9.47	107151.93	–
Serine	340	105.09	60.62	0.13	9.05	39810.72	33884.42
Threonine	290	119.12	76.90	-0.09	8.96	42657.95	34673.69
Tryptophan	520	204.22	143.80	-0.77	9.34	48977.88	34673.69
Tyrosine	220	181.19	–	-0.34	9.04	16595.87	–
Valine	100	117.15	90.75	-0.97	9.52	33113.11	29512.09
